# Case Report: a three-stage treatment strategy combining immunotherapy with chemoradiotherapy for locally advanced esophageal squamous cell carcinoma

**DOI:** 10.3389/fimmu.2026.1800914

**Published:** 2026-05-08

**Authors:** Xiaoyun Zhou, Chunlin Huang, Kexin Ou, Shuhui Xiao, Jiaqi Wang, Jingjing Zhang

**Affiliations:** 1 Department of Radiotherapy, People’s Hospital of Zhongshan, Zhongshan, Guangdong, China; 2 Shenzhen University, Shenzhen, Guangdong, China; 3 The First Clinical Medical College, Guangdong Medical University, Zhanjiang, Guangdong, China

**Keywords:** case report, chemoradiotherapy, esophageal squamous cell carcinoma, immune-related adverse events, immunotherapy

## Abstract

Esophageal squamous cell carcinoma (ESCC), a common malignant tumor with high incidence and mortality in China, remains challenging to treat, especially in advanced stages. Traditional definitive chemoradiotherapy (dCRT) has been the standard care for unresectable locally advanced ESCC but has shown limited long-term efficacy. Recent advances in immune checkpoint inhibitors (ICIs) have opened new avenues for enhancing treatment outcomes. This case report details a 62-year-old male with upper thoracic LA-ESCC (cT3N1M0) who underwent a comprehensive three-stage treatment strategy combining immunotherapy with dCRT. The treatment phases included induction chemotherapy with immunotherapy, followed by radical chemoradiotherapy, and subsequent immunotherapy maintenance. The patient demonstrated significant tumor regression, achieving a complete response and a progression-free survival of 27 months by December 13, 2025. During the treatment, immune-related adverse events (irAEs), including immune diabetes, emerged but were managed effectively through timely intervention and monitoring. This report underscores the potential of immunotherapy combined with dCRT to enhance survival and quality of life in patients with unresectable LA-ESCC. It also identifies key areas for further research: optimizing treatment protocols, improving management of immune-related adverse events, and identifying predictive biomarkers for treatment response. Large-scale prospective clinical trials will be essential to refine these strategies and advance precision therapy in this population.

## 1 Introduction

Esophageal cancer (EC) is the seventh most common malignant tumor and the sixth leading cause of cancer-related deaths worldwide (1). In China, the incidence and mortality rates of EC remain high, and more than 90% of esophageal cancer cases are esophageal squamous cell carcinoma (ESCC) (2). Traditional neoadjuvant or definitive chemoradiotherapy has been the standard treatment for locally advanced ESCC. For unresectable locally advanced ESCC, the standard treatment is dCRT. However, dCRT alone has limited efficacy, with 3-year OS rates for patients ranging from 26.9% to 55.4%, and more than 50% experiencing disease progression within 1 year of treatment (3). Immunotherapy has become an important component of EC treatment, and immune checkpoint inhibitors (ICIs) are now approved for use in first-, second-, and postoperative adjuvant therapy (4–7). The combination of radiotherapy and ICIs is supported by a mechanistic synergy: radiotherapy induces immunogenic cell death, releases tumor antigens, and activates the cGAS-STING pathway, which upregulates PD-L1 expression; while ICIs enhance tumor infiltration and killing of T cells by blocking the PD-1/PD-L1 signaling axis (8). This synergistic effect has demonstrated favorable safety and efficacy profiles in several small-sample studies. To confirm the therapeutic benefits of this combination regimen, multiple large-scale Phase III clinical trials—such as KEYNOTE-975, ESCORT-CRT, RATIONALE 311, and KUNLUN—are currently underway, though their results have not yet been fully reported (9–12).

Despite the remarkable efficacy of immune-combination therapy, the immune-related adverse events (irAEs) still require significant attention. One such event is immune-related diabetes mellitus (irDM), which is a relatively rare but serious endocrine irAEs with an incidence rate of 0.9% (13, 14). While the clinical manifestations resemble traditional diabetes mellitus, irDM has a more acute onset and rapid progression, and requires early recognition and intervention” (13).

Accordingly, this report details a case of locally advanced upper thoracic ESCC (cT3N1M0) in which the patient achieved sustained complete remission (PFS up to 27 months) through a three-phase regimen of “induction chemoimmunotherapy + concurrent chemoradiotherapy + maintenance immunotherapy.” Currently, definitive concurrent chemoradiotherapy is the standard treatment for unresectable locally advanced esophageal squamous cell carcinoma. However, its complete response rate remains unsatisfactory with a high risk of local recurrence. The three-stage strategy adopted in this study has two core objectives: First, the induction phase uses rapid tumor shrinkage by chemotherapy and synergistic effects of immunotherapy to reduce tumor burden and optimize the immune microenvironment before radiotherapy, thereby improving radiosensitivity. Second, the maintenance immunotherapy phase sustains systemic antitumor immune responses triggered by radiotherapy, eliminates residual lesions, and prevents metastasis and recurrence. This strategy aims to break through the limitations of current treatments and improve the chance of long-term cure. This report aims to (1): validate the feasibility and efficacy of this multimodal strategy in such patients (2); focus on discussing the clinical characteristics and successful management experience of irDM that emerged during treatment, to provide key insights for optimizing the clinical practice of combined immunotherapy.

## 2 Case presentation

### 2.1 Initial diagnosis and baseline characteristics (August-September 2023)

**Figure 1:**
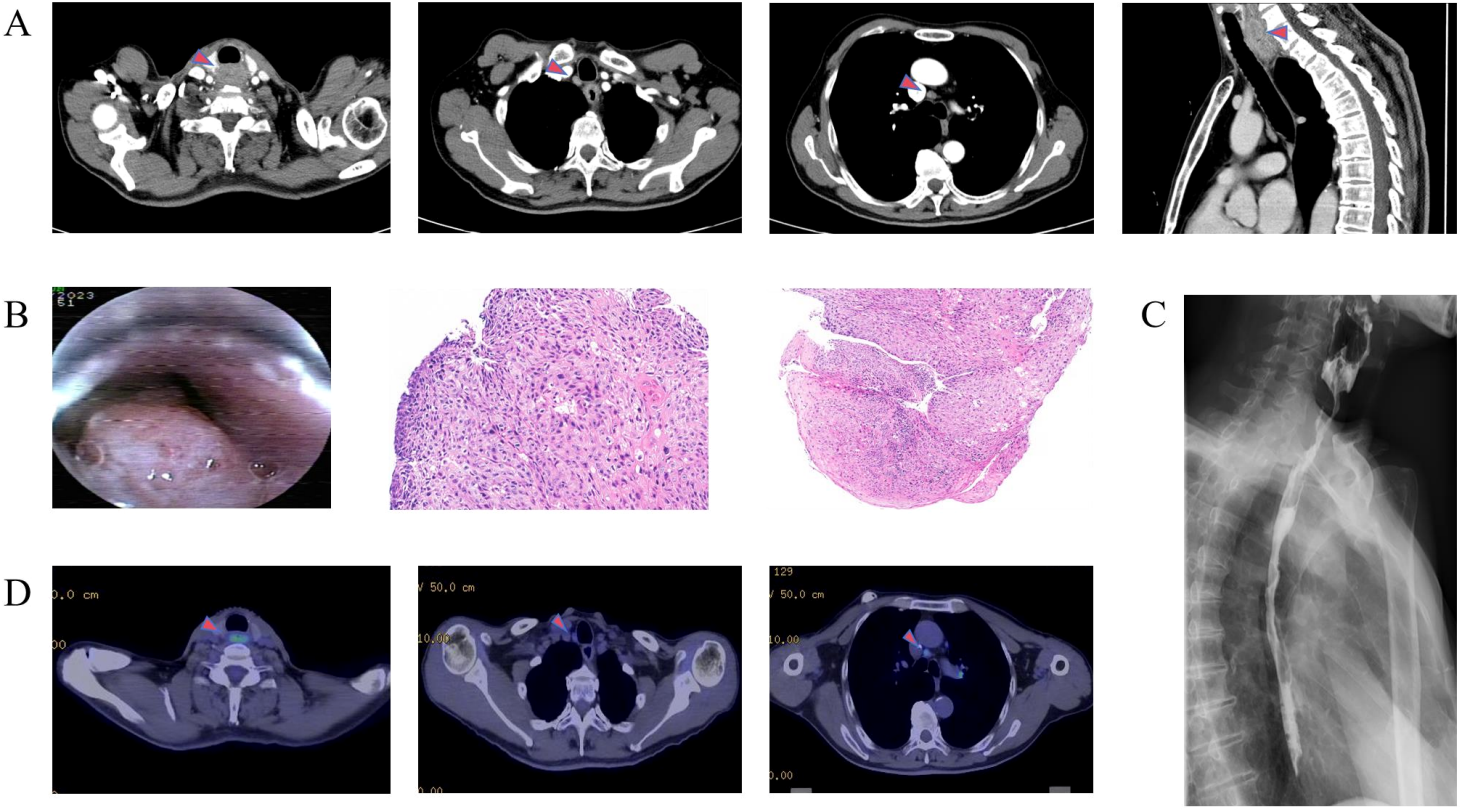
Baseline imaging and histopathology. **(A)** Baseline CT scans (red arrows indicate esophageal mass and lymphadenopathy). **(B)** Esophagoscopy showing upper esophageal mass. Histopathology confirms well-differentiated squamous cell carcinoma (HE × 200). **(C)** Esophagogram with a filling defect in the upper esophagus. **(D)** PET-CT shows hypermetabolism in the upper esophagus and mediastinal nodes (red arrows).

A 62-year-old male patient presented in August 2023 with a chief complaint of progressive dysphagia for three months. Baseline assessment revealed an ECOG Performance Status (PS) of 1, a Body Mass Index (BMI) of 18.8 kg/m², and a Nutritional Risk Screening 2002 (NRS2002) score of 2, indicating nutritional risk. Laboratory investigations at diagnosis revealed results within normal reference ranges across multiple parameters: tumor marker profiles, including carcinoembryonic antigen (CEA), squamous cell carcinoma antigen (SCC), cancer antigen 125 (CA-125), and carbohydrate antigen 19-9 (CA19-9); fasting plasma glucose (6.1 mmol/L); and thyroid function tests comprising free triiodothyronine (FT3), free thyroxine (FT4), and thyroid-stimulating hormone (TSH). Imaging and pathological examinations confirmed the diagnosis of upper thoracic esophageal squamous cell carcinoma (ESCC). A water-soluble contrast esophagogram showed a filling defect approximately 4.6 cm in length at the C6-T2 level (**Figure 1C**). Esophagogastroduodenoscopy (EGD) identified an elevated, protruding mass involving the esophageal mucosa 15–22 cm from the incisors (**Figure 1B**). Histopathological examination of the biopsy specimens confirmed well-differentiated squamous cell carcinoma (**Figure 1B**). Further evaluation with contrast-enhanced chest computerized tomography (CT) showed an 11 mm thickened wall with adjacent enlarged mediastinal lymph nodes (**Figure 1A**). Staging work-up, including cranial magnetic resonance imaging (MRI) and whole-body bone scintigraphy, showed no evidence of distant metastasis. The patient was diagnosed with locally advanced ESCC cT3N1M0, Stage III, according to the American Joint Committee(AJCC) on Cancer, 8th Edition staging system.

### 2.2 Induction chemoimmunotherapy and response evaluation (September-October 2023)

**Figure 2:**
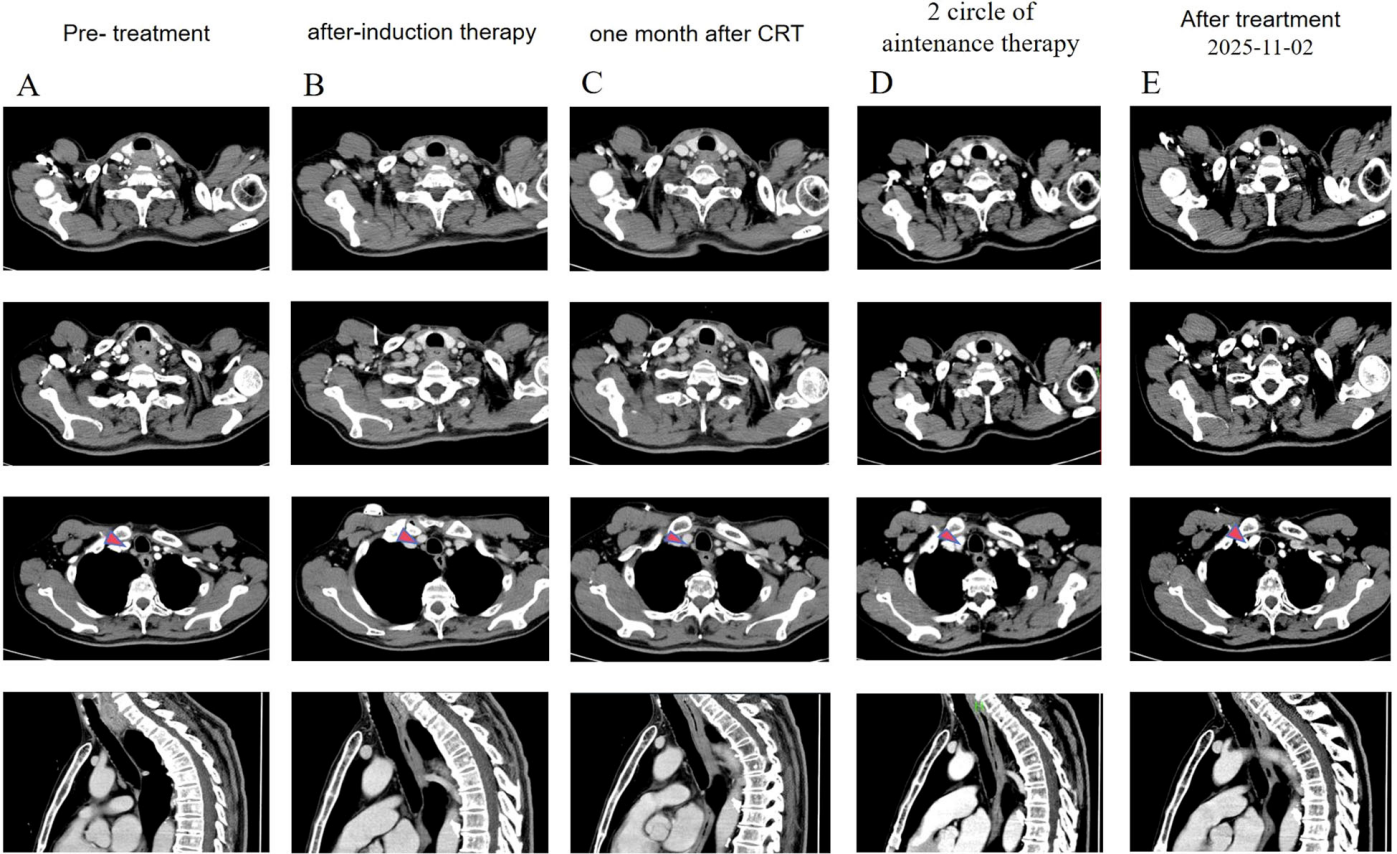
Images from pre- and post-treatment. **(A)** Pre-treatment baseline contrast-enhanced chest CT (axial and sagittal views). **(B)** Restaging after 2 cycles of induction therapy. **(C)** Follow-up 1 month after definitive concurrent chemoradiotherapy. **(D)** Restaging after 2 cycles of maintenance immunotherapy. **(E)** Recent follow-up after completion of therapy.

With an upper thoracic tumor at clinical stage cT3N1M0, the patient was not considered a surgical candidate, and his nutritional status was not suitable for immediate radiotherapy. On September 13, 2023, the patient commenced the first cycle of a 21-day induction therapy regimen, which comprised two total cycles. The pharmacological intervention consisted of the following protocol: liposomal paclitaxel (175 mg/m²) administered via intravenous infusion on day 1 of each cycle; cisplatin (75 mg/m²) delivered in divided doses on days 1–3 of each cycle; and tislelizumab (200 mg) administered via intravenous infusion on day 1 of each cycle. Following two cycles of induction therapy, follow-up evaluation with contrast-enhanced chest CT and esophagogastroduodenoscopy (EGD) demonstrated a treatment response: esophageal wall thickening was reduced to 8 mm (previously 11 mm, **Figure 2B**), and the filling defect in the upper esophagus decreased 4.1 cm(previously 4.6 **cm**) in length, with concomitant widening of the luminal stenosis 0.7 cm(previously 0.4 cm). Subsequent Positron Emission Tomography-Computed Tomography (PET-CT) indicated residual metabolic activity in the cervical esophagus (SUVmax 10.5, **Figure 1D**) and reactive hyperplasia of mediastinal lymph nodes (SUVmax 3.4, **Figure 1D**). Overall, the therapeutic response was assessed as a partial response (PR) according to the Response Evaluation Criteria in Solid Tumors (RECIST) version 1.1.

### 2.3 Definitive chemoradiotherapy combined with immunotherapy and response (November-December 2023)

**Figure 3:**
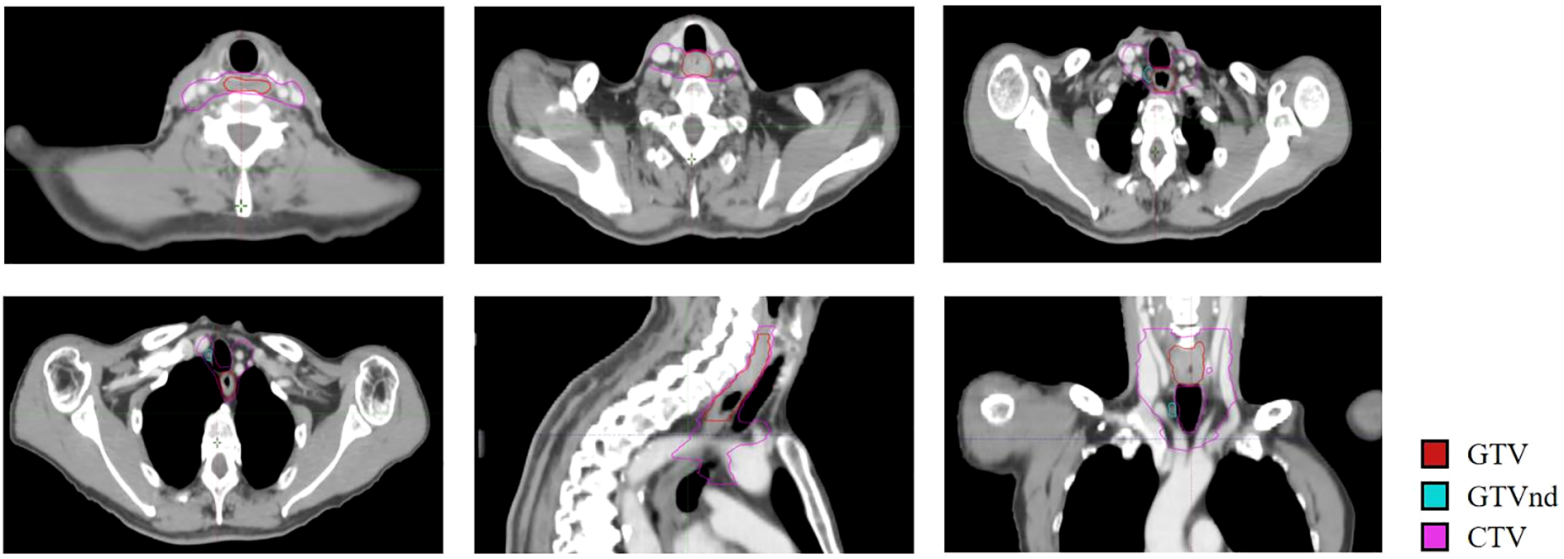
Delineation of radiotherapy target volumes. Planning CT images in multiple planes (axial, coronal, and sagittal) is shown. The gross tumor volume (GTV, red), the nodal gross tumor volume (GTVnd, cyan), and the clinical target volume (CTV, purple) are delineated. A color legend is provided.

Following the completion of induction therapy, the patient underwent definitive concurrent chemoradiotherapy from November 13 to December 20, 2023. Radiotherapy was delivered using intensity-modulated radiation therapy (IMRT) with the following prescription doses: 60 Gy in 28 fractions to the gross tumor volume (GTV), and 50.4 Gy in 28 fractions to both the clinical target volume (CTV) and the metastatic lymph node volume (GTVnd). The concurrent chemotherapy regimen consisted of liposomal paclitaxel (50 mg/m²) and cisplatin (25 mg/m²), administered weekly via intravenous infusion. The detailed delineation of the target volume is presented in **Figure 3**. Concurrent immunotherapy with tislelizumab (200 mg) was continued every three weeks throughout this phase. One month after completing radiotherapy (January 15, 2024), a follow-up enhanced chest CT and esophagogram showed further improvement: an esophageal wall thickness of 7mm(previously 8 mm, **Figure 2C**) with the filling defect shortened to 2.9 cm(previously 4.6 cm) and the lumen widened to 0.8 cm(previously 0.4 cm). The response was maintained as a PR.

### 2.4 Maintenance immunotherapy, adverse event management, and long-term outcome

After the completion of concurrent chemoradiotherapy, maintenance treatment with single-agent tislelizumab was initiated on January 17, 2024, at a dose of 200 mg every 3 weeks until disease progression or the occurrence of intolerable toxic reactions.

#### 2.4.1 Maintenance therapy and tumor response

Maintenance therapy with tislelizumab monotherapy (200 mg every 3 weeks) was started on January 17, 2024. A follow-up chest CT on March 22, 2024, showed that the esophageal wall thickness had decreased to approximately 0.5cm(previously 0.7cm, **Figure 2D**) within the normal range. After completing 7 cycles, maintenance immunotherapy was discontinued in June 2024 due to the onset of immune-related adverse events (irAEs).

#### 2.4.2 Immune-related hypothyroidism

During the maintenance immunotherapy phase, the patient sequentially developed two distinct endocrine immune-related adverse events (irAEs). In June 11, 2024 (corresponding to the seventh cycle of maintenance immunotherapy), significant thyroid dysfunction was observed, characterized by a elevation of thyroid-stimulating hormone (TSH) to 97.54 μIU/mL (reference range: 0.55-4.78 μIU/mL), accompanied by a decrease in free thyroxine (FT4) to 6.55 pmol/L (reference range: 11.5-22.7 pmol/L) and free triiodothyronine (FT3) to 2.95 pmol/L (reference range: 3.5-6.5 pmol/L). Based on these findings, a diagnosis of immune-mediated thyroiditis with primary hypothyroidism was established, and levothyroxine replacement therapy was initiated. It is noteworthy that the occurrence of immune-mediated thyroid injury is recognized as a potential predictor for the subsequent development of immune checkpoint inhibitor-induced diabetes mellitus.

#### 2.4.3 Immune-related diabetes mellitus

On May 20, 2024, during the sixth cycle of maintenance immunotherapy, the patient initially presented with an elevated fasting blood glucose of 11.48 mmol/L that remained untreated. Subsequently, on June 11, 2024, he developed acute symptoms of polydipsia and polyuria prompting medical evaluation. Laboratory investigations revealed significant hyperglycemia (random glucose: 29.14 mmol/L) with an HbA1c of 11.5%. Metabolic testing showed mildly elevated beta-hydroxybutyrate (0.35 mmol/L), indicating a tendency toward diabetic ketosis, while pancreatic enzymes (amylase and lipase) remained within normal ranges. Abdominal CT imaging demonstrated no pancreatic abnormalities. Notably, the patient’s pre-immunotherapy HbA1c was 5.2% (normal range <5.7%), and there was no personal or family history of diabetes mellitus. A markedly low C-peptide level (0.37 ng/mL) confirmed absolute insulin deficiency, leading to a diagnosis of irDM. Management involved the immediate and permanent discontinuation of tislelizumab. Acute hyperglycemia was rapidly stabilized using a continuous subcutaneous insulin infusion (insulin pump). Upon achieving glycemic stability, the regimen was transitioned to a combination of degludec/aspart insulin and metformin, with subsequent fine-tuning of insulin dosages guided by dynamic glucose monitoring. With standardized management, glycemic control was optimized, resulting in a follow-up blood glucose level of 7.14 mmol/L by December 2025.

#### 2.4.4 Long-term follow-up and final outcome

**Figure 4:**
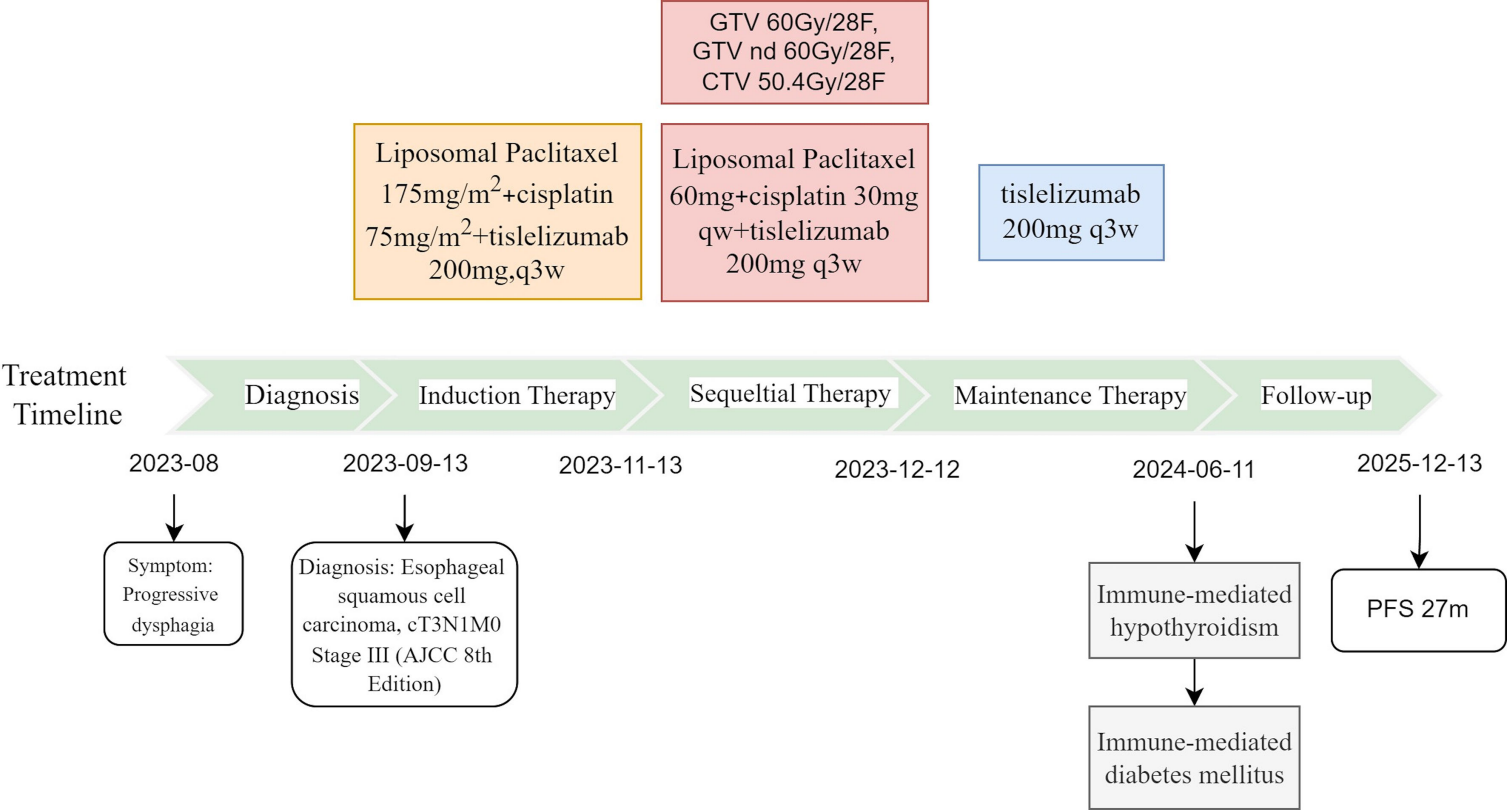
The treatment timeline.

The patient’s entire antitumor treatment course and medication details are illustrated in **Figure 4**. As of the last follow-up on December 13, 2025, the most recent imaging studies (contrast-enhanced chest CT, esophagogram) showed a normal esophageal wall thickness (3 mm, **Figure 2E**) with no evidence of residual tumor or lymph node metastasis. The response was confirmed as a complete response (CR). From the time of diagnosis in September 2023, the patient achieved a progression-free survival (PFS) exceeding 27 months. The ECOG PS score was 0, symptoms of dysphagia had completely resolved, allowing a normal diet, and the patient maintained a good quality of life.

## 3 Discussion

The three-stage sequential combined immunotherapy strategy proposed in this study aims to address the core clinical challenge of high recurrence rates after definitive concurrent chemoradiotherapy for locally advanced esophageal squamous cell carcinoma. By advancing immunotherapy to the induction phase and conducting maintenance after dCRT, this protocol constructs an integrated “induction-concurrent-consolidation/maintenance” model to overcome the therapeutic plateau of conventional dCRT. Objectively, dCRT remains the standard curative modality for this population, achieving complete response (CR) in some patients. The profound and durable remission in this case is primarily attributed to dCRT’s core antitumor efficacy. On this basis, 2-cycle induction chemoimmunotherapy achieved partial response (PR), indicating it could rapidly reduce tumor burden for subsequent radiotherapy and potentially reverse the immunosuppressive microenvironment to enhance radiosensitivity. Post-dCRT maintenance immunotherapy further consolidated efficacy, eradicated minimal residual disease, and established long-term immunologic memory, consistent with the PACIFIC consolidation paradigm in lung cancer. Additionally, favorable patient characteristics—relatively young age (62 years), good baseline performance status (ECOG PS 1), and excellent treatment tolerance—were crucial for completing the intensive regimen and gaining significant benefit. Thus, the patient’s long-term complete remission likely results from the combined effects of standard dCRT, sequential immunotherapeutic intensification, and advantageous patient factors. This case demonstrates the clinical feasibility of this comprehensive management model, rather than asserting the absolute superiority of any single component.

Immune checkpoint inhibitors (ICIs) have reshaped the therapeutic landscape for esophageal cancer, offering new hope for patients with unresectable locally advanced disease. Among various approaches, the combination of ICIs with definitive chemoradiotherapy demonstrates unique advantages. The synergistic mechanism lies in the ability of radiotherapy to induce immunogenic cell death, releasing tumor antigens, and activating systemic anti-tumor immunity through pathways such as cGAS-STING, exerting an “*in situ* vaccine” effect (14–18). However, radiotherapy can also trigger adaptive immunosuppression, particularly by upregulating PD-L1 expression, leading to T-cell exhaustion, which may impact the abscopal effect (19–22). The immunogenic microenvironment created by neoadjuvant chemoradiotherapy, combined with the immune suppression reversal action of ICIs, theoretically produces a “1 + 1>2” synergistic effect, potentially enhancing primary tumor regression and activating systemic immune responses to control distant metastases (23).

In recent years, multiple studies have shown that immunotherapy combined with definitive chemoradiotherapy can improve survival rates in patients with locally advanced esophageal cancer. A single-arm phase II trial (EC-CRT-001, NCT04005170) in unresectable locally advanced esophageal squamous cell carcinoma evaluated the efficacy and safety of toripalimab combined with concurrent chemoradiotherapy (8). The study enrolled 42 patients, treated with definitive concurrent chemoradiotherapy (paclitaxel + cisplatin, weekly for 5 cycles) combined with toripalimab (240 mg every 3 weeks). Immunotherapy maintenance continued for up to 1 year, or until disease progression or intolerable toxicity. Results showed a complete response rate of 62.0% with the combination, superior to historical data (31.0%–56.0%); the 1-year overall survival and progression-free survival rates were 78.4% and 54.5%, respectively. The safety profile was manageable, with the main adverse events being lymphopenia (36 cases) and leukopenia (8 cases). Another single-arm phase II study (ImpactCRT, ChiCTR2000034304) further confirmed the outstanding efficacy of the “induction immunotherapy plus chemotherapy followed by sequential concurrent chemoradiotherapy” model in locally advanced unresectable esophageal squamous cell carcinoma (24). This study enrolled 42 patients who received 2 cycles of induction therapy (albumin-bound paclitaxel + carboplatin + camrelizumab) followed by sequential concurrent chemoradiotherapy (fluorouracil + cisplatin). The objective response rate reached 91.3%, with 1-year overall survival and 1-year progression-free survival rates of 87.0% and 71.7%, respectively, significantly better than historical data for traditional definitive chemoradiotherapy, providing high-level evidence for the forward shift of immunotherapy. Building on the consolidation model established by the PACIFIC trial in non-small cell lung cancer, a similar potential is emerging in esophageal squamous cell carcinoma (25). The latest data from a single-arm phase II study by Professor Jun Wang’s team (ASCO 2025) showed that among 32 patients with unresectable locally advanced esophageal squamous cell carcinoma who received camrelizumab consolidation therapy after definitive chemoradiotherapy, with a median follow-up of 25.1 months, the median progression-free survival and overall survival were not reached. The 1-year and 2-year progression-free survival rates were 81.3% and 60.6%, respectively, and the overall survival rates were 96.9% and 81.0%, with a disease control rate of 59.4%. Most adverse events were grade 1-2, with no grade 4–5 events reported; pneumonitis occurred in 31.3% of patients (all grade 1-2) (26). These results highlight the significant value of immune consolidation in the treatment of esophageal cancer.

Compared with the above pivotal clinical studies, this case adopted a full three-stage combined regimen: induction chemoimmunotherapy + concurrent chemoradiotherapy + maintenance immunotherapy, which is more individualized and clinically innovative in treatment design. In contrast to the EC-CRT-001 study that directly used concurrent chemoradiotherapy combined with immunotherapy, this case received upfront induction chemoimmunotherapy, which rapidly reduced tumor burden, relieved esophageal obstruction, and improved nutritional status, significantly enhancing treatment tolerance in this elderly patient at nutritional risk. Different from the ImpactCRT study without standardized maintenance immunotherapy, this case completed a full-course of maintenance immunotherapy after radical chemoradiotherapy, providing crucial support for achieving long-term complete response. As a single case report, this study has certain limitations. Large-sample prospective clinical trials are still needed in the future to further validate the efficacy and clinical safety of this combined regimen.

However, immune-related adverse events (irAEs) often lead to treatment interruption or delay. The long-term remission maintained in this patient following tislelizumab discontinuation due to immune-related adverse events (irAEs) suggests that immune checkpoint inhibitors may induce durable anti-tumor immune memory. The underlying mechanisms likely involve two key aspects. First, immunotherapy activates and expands tumor-specific T cells, enabling targeted recognition and elimination of cancer cells. Due to the inherent memory properties of immune cells, a sustained response can persist even after treatment cessation, translating into significant survival benefits. This process may involve the effective conversion of effector T cells into long-lived memory T cells, thereby establishing persistent immune surveillance that suppresses tumor recurrence (27, 28). Second, evidence indicates that some patients who discontinue treatment because of severe irAEs can exhibit deeper and more durable tumor regression. This suggests that the onset of irAEs may reflect a broadly activated and potentiated immune system. Such systemic immune activation may persist after drug withdrawal, resulting in a unique clinical phenomenon described as a “side effect accompanied by benefit” effect (29).

A pooled analysis of trials involving nivolumab combined with ipilimumab by Schadendorf, Horiguchi et al. showed that patients who discontinued treatment due to irAEs had comparable efficacy to those who continued, with similar complete response rates and duration of response; furthermore, the estimated survival of patients who discontinued was even longer (30, 31). An analysis of 2794 patients across two centers by Mark Awad’s team also indicated that a longer duration of treatment before discontinuation was associated with better outcomes after discontinuation (P<0.001) (32). This suggests that even with early discontinuation of immunotherapy, especially for patients with high PD-L1 expression or those who have already achieved a treatment response, durable efficacy may still be obtained. Further research is needed to optimize decisions regarding treatment duration, balancing efficacy and toxicity.

Immune-related diabetes mellitus exhibits unique clinical features compared with type 1 and type 2 diabetes mellitus, which is critical for early identification. irDM is characterized by acute onset and rapid progression, similar to type 1 diabetes, but is usually negative for diabetes-related autoantibodies including anti-GAD, anti-IA2, anti-insulin, and anti-ZnT8 antibodies, accompanied by significantly decreased C-peptide levels, indicating absolute insulin deficiency (33, 34). In contrast, classic type 1 diabetes is positive for relevant autoantibodies in most cases (35), whereas type 2 diabetes is featured by insidious onset, relatively preserved islet function, and rare ketoacidosis. In the present case, the patient presented with acute polydipsia, polyuria, and severe hyperglycemia, negative diabetes-related autoantibodies, markedly low C-peptide level, and a tendency to ketosis, which was fully consistent with the typical features of irDM. Notably, the patient had previously developed immune-mediated hypothyroidism, a well-recognized precursor of irDM, further supporting the diagnosis of immune-related endocrine adverse event rather than primary diabetes (36, 37). Unlike some other immune-related adverse events, irDM is refractory to glucocorticoid therapy due to the typically irreversible destruction of pancreatic β-cells, resulting in absolute insulin deficiency. Therefore, once diagnosed, immediate and lifelong insulin replacement therapy is required. As illustrated in this case, glycemic stability was achieved through transitioning from an insulin pump to a basal-bolus insulin regimen (38).

Although irDM has a relatively low incidence, it can lead to serious clinical consequences. The clinical presentation of irDM resembles that of classic type 1 diabetes, and it is frequently accompanied by ketoacidosis—with reported incidence rates as high as 40%–76%—which often serves as the initial manifestation of the condition. The time of onset varies widely, with a median of approximately 7–17 weeks, often occurring around the fourth treatment cycle (13, 14, 39–41). The pathogenesis involves autoimmune attack: blockade of the PD-1/PD-L1 pathway leads to the activation of autoreactive T cells, destroying pancreatic beta cells and causing absolute insulin deficiency (approximately 92% of patients have low C-peptide levels at onset) (42, 43). Additionally, the risk is significantly increased in carriers of the DR4-DQ8 or DR3-DQ2 haplotypes, with the prevalence of Human Leukocyte Antigen-DR4 (HLA-DR4) in patients with ICI-associated diabetes even higher than in classic type 1 diabetes, making it a potential prospective predictive marker (44, 45). Therefore, combining attention to the treatment cycle (especially around the fourth cycle), monitoring C-peptide levels, vigilance for signs of ketoacidosis, and screening patients for high-risk HLA-DR4 haplotypes can aid in the early identification and prediction of the risk of this complication.

While this case achieved significant survival benefit (PFS exceeding 27 months) and maintained a good quality of life, it was also complicated by irDM requiring lifelong insulin therapy. This outcome underscores the double-edged nature of intensive combination immunotherapy. As an irreversible endocrine toxicity, irDM imposes lifelong disease management burdens, affects daily quality of life, and carries potential risks of long-term microvascular and macrovascular complications. This case highlights that, while pursuing survival gains, vigilance against serious immune-related adverse events and the establishment of proactive monitoring systems are essential. Based on the findings of this case and existing evidence, future research may integrate high-risk HLA-DR4 haplotypes, immunotherapy cycles, C-peptide levels, and prior immune-related thyroid injury to establish a risk prediction and stratification model for immune-related diabetes in patients with unresectable locally advanced esophageal squamous cell carcinoma. This model can be used for pre-treatment screening of high-risk populations and dynamic monitoring of blood glucose and islet function during treatment, enabling early warning and individualized prevention of severe endocrine immune-related adverse events, thereby improving the safety and practicability of immunotherapy combined with chemoradiotherapy.

## 4 Conclusion

In conclusion, we report a case of unresectable locally advanced ESCC that was successfully treated with an innovative three-stage strategy integrating immune checkpoint inhibitors (ICIs) with definitive chemoradiotherapy (dCRT), achieving a durable complete response. This case suggests that immunotherapy may hold significant potential for improving outcomes of conventional chemoradiotherapy. Notably, sustained antitumor efficacy was maintained even after treatment discontinuation due to severe immune-related adverse events, such as immune-related diabetes. Our experience underscores the necessity for close monitoring and management of endocrine immune-related adverse events. Though the results are encouraging, the nature of single-case studies calls for cautious interpretation. Moving forward, this case highlights the need to validate such integrated approaches in prospective trials, define optimal therapeutic sequencing, and identify biomarkers(PD-L1, TMB, MSI, etc.) to better predict response and toxicity, thereby guiding more personalized treatment strategies.

## Data Availability

The original contributions presented in the study are included in the article/supplementary material. Further inquiries can be directed to the corresponding author.
